# Where Are We with RPE Replacement Therapy? A Translational Review from the Ophthalmologist Perspective

**DOI:** 10.3390/ijms23020682

**Published:** 2022-01-08

**Authors:** Raffaele Raimondi, Piero Zollet, Francesco Paolo De Rosa, Panagiotis Tsoutsanis, Matteo Stravalaci, Marianna Paulis, Antonio Inforzato, Mario R. Romano

**Affiliations:** 1 IRCCS Humanitas Research Hospital, Via Manzoni 56, 20089 Rozzano–Milan, Italy; piero.zollet@humanitas.it (P.Z.); Matteo.stravalaci@humanitasresearch.it (M.S.); marianna.paulis@humanitasresearch.it (M.P.); antonio.inforzato@humanitasresearch.it (A.I.); 2 Department of Biomedical Sciences, Humanitas University, Via Rita Levi Montalcini 4, 20072 Pieve Emanuele–Milan, Italy; derosafrancescopaolo@gmail.com (F.P.D.R.); tpanos02@gmail.com (P.T.); mario.romano@hunimed.eu (M.R.R.); 3 Institute of Genetic and Biomedical Research (IRGB), UOS of Milan, National Research Council of Italy, 20138 Milan, Italy; 4 Eye Center, Humanitas Gavazzeni-Castelli, 24128 Bergamo, Italy

**Keywords:** retinal pigmented epithelium, replacement, induced pluripotent stem cells, embryonic stem cells, age related macular degeneration, Stargardt disease

## Abstract

The retinal pigmented epithelium (RPE) plays a pivotal role in retinal homeostasis. It is therefore an interesting target to fill the unmet medical need of different retinal diseases, including age-related macular degeneration and Stargardt disease. RPE replacement therapy may use different cellular sources: induced pluripotent stem cells or embryonic stem cells. Cells can be transferred as suspension on a patch with different surgical approaches. Results are promising although based on very limited samples. In this review, we summarize the current progress of RPE replacement and provide a comparative assessment of different published approaches which may become standard of care in the future.

## 1. Introduction

### 1.1. RPE and BRB’s Anatomy and Physiology

The retinal pigment epithelium (RPE) is a single cell layer composed of hexagonal polarized cells with microvilli that extend from the apical surface to envelop the outer segments of rods and cones of the photoreceptor (PR) layer of the retina. The RPE plays a central role in vision because of both physical and metabolic reasons [1,2].

What makes it a pigmented epithelium is the presence of melanosomes containing light-absorbing melanin, which, by absorbing the light that passes through the PR layer, prevents it from being reflected on the PR itself. In the absence of melanin, the visual image would be degraded.

The RPE plays a central role in the visual cycle by converting all-trans-retinol back to 11-cis retinal, especially in cones.

Photoreceptors contain high amounts of photosensitive molecules, which accumulate in the form of photo-damaged proteins and lipids. In addition, the retina itself can generate photo-oxidative radicals. For this reason, the photoreceptor outer segments (POS) regenerate themselves by shedding their tips and reform from their bases. The shed POS are eventually phagocytosed by the RPE.

Moreover, the RPE is essential to maintain subretinal homeostasis through the transport of nutrients, ions, fluids, metabolic end products, and production of several growth factors, including pigment epithelium-derived factor (PEDF) and vascular endothelial growth factor (VEGF). The RPE is indeed a component of the outer blood retinal barrier (oBRB), which, together with the inner blood retinal barrier (iBRB), constitutes the blood retinal barrier (BRB). The BRB provides a suitable, highly regulated, chemical environment for the avascular transparent tissues of the eye as well as serving as a drainage route for the waste products of the metabolic activity of the ocular tissues.

The iBRB is established by tight junctions (TJs) (zonulae occludentes) between neighboring retinal endothelial cells, and rests on a basal lamina that is covered by the processes of astrocytes, Muller cells, and pericytes. The function of these cellular processes is to regulate the activity of the retinal endothelial cells and the iBRB by transmitting regulatory signals to the endothelial cells that convey information on the microenvironment of the retinal neuronal circuitry.

Proximally to the inner layers of the retina, the oBRB is established by the TJs of neighboring RPE cells. The layer of RPE cells is found adjacent to the Bruch’s membrane which separates the neural retina from the fenestrated choriocapillaris, and its function is to regulate access from the blood to the photoreceptors while eliminating waste products.

In both iBRB and oBRB, the cell’s TJs restrict the paracellular movement of fluids and molecules between blood and retina, and the endothelial cells and RPE cells actively regulate inward and outward movements. This allows the levels of amino acids and fatty acids within the retina to be kept relatively stable no matter how much these levels fluctuate in the blood.

The RPE is involved in multiple degenerative retinal pathologies, mainly in age-related macular degeneration (AMD) and retinitis pigmentosa. Thus, there is a great interest in it as a possible therapeutic target [3].

### 1.2. RPE and Age-Related Macular Degeneration

AMD pathophysiology is a complex interaction of intricated and poorly characterized mechanisms. Nonetheless, several studies point to the retinal pigmented epithelium (RPE) as an important player in AMD pathogenesis [1,2].

In this regard, the oxidative stress hypothesis highlights that the imbalance between oxidants and anti-oxidant systems in the retinal microenvironment leads to accumulation of reactive oxygen and nitrogen species, and these trigger and sustain chronic inflammation [4,5]. In conditions of oxidative damage, the RPE becomes dysfunctional and, in severe cases, necrotic, which in turn enhances drusen deposits and complement system activation and inflammation [6,7,8,9,10]. Furthermore, this can cause intercoupling of melanin with lipofuscin, pigment granules composed of lipid-containing residues of lysosomal digestion, which generate reactive oxygen species upon excitation with blue light, thereby making the aged RPE more susceptible to oxidative damage. Oxidative stress is indicated also by the accumulation of advanced-glycation end products (AGEs) in RPE and BrMb (drusen also contain AGEs) [11].

Finally, chronic oxidative stress impairs autophagy and therefore causes the accumulation of lipofuscin and drusen formation [12].

Another mechanism that induces AMD is via an impairment in the choroidal perfusion, which can potentially cause ischemia of the RPE and thus lead to AMD [11,12,13]. The age-related remodeling of Bruch’s membrane leads to the accumulation of drusen bodies. These soft drusen, and associated basal laminar and linear deposits, interfere with the outer BRB by impeding diffusion between the choriocapillaris and RPE. The consequential reduction in permeation of amino acids, fluids, metabolites, and proteins across the membrane has very significant negative consequences on the RPE layer’s function [13]. Lastly, senescence of RPE cells results in a senescence-associated secretory phenotype that causes progression of AMD [13,14].

### 1.3. RPE and Stargardt Disease

Stargardt disease (STGD1) is the most common inherited macular dystrophy in both adults and children with a prevalence of 1 in 8000–10,000, also presenting a very wide clinical and genetic spectrum [3,14,15].

In STGD1, the loss of function of ABCA4 resulting in the disruption of the visual cycle eventually causes the accumulation of lipofuscin and its by-products. According to current literature, lipofuscin and its components, especially N-retinylidene-N-retinylethanolamine (A2E), are toxic to the RPE, thus damaging it, potentially leading to secondary photoreceptor degeneration [15].

Clinical presentation in early phases may reveal a normal fundus or mild RPE anomalies. Diagnosis at this point can be delayed if further testing is not prompted. Imaging with fundus auto fluorescence (FAF) can be useful to screen suspicious cases. In the early phases, lipofuscin deposition and RPE stress can be detected by an increase in autofluorescence. Progressively, with degeneration of RPE, there is the appearance of areas of atrophy extending from the fovea, macula, and eventually expanding to the whole posterior pole. RPE atrophy is seen as dark patches at FAF. Confirmation of diagnosis can be done by electrophysiology and genetic testing [16].

Disease severity and progression according to different studies correlates well with the degree of extension of the atrophic RPE patch at FAF. Disease severity and progression according to different studies correlates well with the degree of extension of the atrophic RPE patch at FAF [16].

### 1.4. Cell Therapies of the RPE

The human retina is a complex structure made up of multiple layers of cells with different features and functions. Retinal innermost layer is made up by a complex circuitry of neuronal cells which includes retinal ganglion cells, bipolar cells, amacrine cells, and PRCs.

PRCs are located in the most external layer of the so called neuroretina and are in close anatomical relation with RPE.

The RPE supports metabolically and functionally the PRCs. Therefore, when RPE degenerates, as happens in AMD, ultimately the PRCs degenerate as well alongside them.

In theory, there are multiple strategies to prevent the aforementioned process, however only a few proved feasible and have been more extensively tested than others.

Potentially, one can choose to either support and rescue the damaged retinal cells or to replace them. Cell rescue can possibly be pursued by providing retinal cells with trophic cytokines and factors or by immunomodulation [17,18,19]. Cell replacement instead aims to repair the damaged tissue by providing new support cells to the neuroretina [20].

It is important to highlight that cell replacement might act only by providing indirect support for host tissue through nutrients or growth factors. In fact, it has not been demonstrated that grafted RPE develop functional interactions with host photoreceptors.

Restoring the inner retinal layers and their complex network is challenging once atrophy has ensued. In contrast, preventing PRC degeneration by restoring an RPE layer before it is too late is in our opinion a more feasible task.

With these concepts in mind, it is quite apparent why researchers’ approaches to the treatment of AMD are strongly focused on the replacement of RPE cells and on the development of stem cell-based strategies in order to restore the damaged RPE layer and the functionality of the blood–retinal barrier which these cells are a part of. On the other hand, Stargardt disease is caused by a PRC mutation, but may however benefit from this therapeutical approach.

These cutting-edge intervention strategies are presented and discussed in the following sections, with a primary focus on clinical trials conducted with human induced pluripotent stem cells (hiPSCs) and embryonal stem cells (hESCs) as sources of RPE progenitors.

## 2. Results

### 2.1. Dry AMD

Schwartz et al. described the outcomes of nine AMD patients with GA treated in phase I/II study (median age 77 years old) [21,22]. The patients were treated with a ESC-derived RPE cellular suspension obtained from a hESC line (MA09), delivered into the subretinal space with a 38G cannula at the transition zone between healthy and atrophic retina after 25G vitrectomy [23]. Patients underwent systemic low dose immunosuppression 1 week before surgery and up to 12 weeks afterwards. Authors report no adverse event related to the cells, 1 patient developed a mild epiretinal membrane, 1 patient developed a vitreous inflammation that resolved after 2 months with antibiotic injection, and 1 eye developed cataract that required surgery. Best corrected visual acuity (BCVA) at 6 months improved by at least 15 letters in 4 eyes, 11 to 14 letters in 2 eyes, and remained stable in 3 eyes (change of <10 letters) [24]. Authors report no correlation between the presence of postoperative pigmentation and postoperative visual improvement, nor did the absence of hyperpigmentation preclude visual improvement [24].

Riemann et al. published intermediate results of a Phase I/IIa clinical study in patients with dry AMD and GA [25]. In this study, subretinal injection of hESCs was performed. The injection was carried out either via pars plana vitrectomy (PPV) and retinotomy or with the Orbit subretinal delivery system (SDS) under local anesthesia. Using PPV, the most common ocular adverse events (AEs) were the formation of 2 cases of epiretinal membrane (ERM) that required surgical peeling and 1 retinal detachment. AEs associated with the Orbit SDS include eyelid edema and subconjunctival hemorrhage. A visual improvement of 10 to 22 letters has been reported in the last cohort treated.

Kashani et al. carried out a study using a patch of hESC-derived RPE on a Parylene C scaffold enrolling 5 AMD patients with GA [26]. The patch was obtained through a multi-step engineering process that led to a layer of cells displaying RPE markers (e.g., RPE65) as well as phagocytosis of photoreceptor outer segments with a minimum density of 10^5^ cells per scaffold [23]. The patch was implanted in 4 patients. No AE were reported and 1 patient improved by 17 letters (Table 1).

### 2.2. Wet AMD

Tezel et al. in 2007 transplanted allogenic RPE that was obtained from donors within 24 h of death in 12 eyes of 12 patients [27]. All 12 patients were treated with triple immune suppression (corticosteroids, azathioprine, cyclophosphamide) preoperatively and postoperatively. Graft fibrosis was seen in 6 patients who stopped the immune suppression. AE reported cataract progression requiring surgery (3 of 8 phakic eyes), retinal detachment (3 eyes), intraoperative retinal breaks (2 eyes), and macular pucker (2 eyes).

Binder et al. enrolled in their trial 53 patients, of which 39 patients underwent RPE cells transplantation along with membranectomy while 14 patients underwent membranectomy only as a control group [28]. RPE cells were harvested subretinally from the nasal side of the optic disc, centrifugated and then injected into the subretinal space. Authors reported significantly better reading acuity and higher multifocal electroretinogram (mfERG) responsedensity than control subjects.

Mandai et al. reported one case of autologous RPE implant obtained from iPS-derived from skin fibroblasts [29]. The patient underwent subfoveal CNV excision during the same PPV, and no scaffold was used. One year after surgery, the transplanted sheet remained intact, and BCVA was stable without the need for anti-VEGF injections.

Da Cruz et al. enrolled two AMD patients with subfoveal CNV and subretinal hemorrhage [30]. The authors starting from hESC obtained a 6 mm × 3 mm patch of a well differentiated RPE monolayer laying on a vitronectin-coated polyester membrane that was transplanted into the subretinal space and placed under the macula. AEs included one retinal detachment requiring surgery. BCVA improved respectively with 29 and 21 ETDRS letters along with structural evidence of RPE patch integration and focal improvement in the photoreceptor anatomy over the transplant.

Sugita et al., with the purpose of minimizing immune reactions, developed a cellular suspension of hiPS-derived RPE cells obtained from HLA-homozygous haplotypes matching those of patients with exudative AMD [26]. The transplant was performed in 5 patients with exudative AMD. Main AE were two initial suspected rejections controlled with pharmacotherapy and 1 ERM. No change in BCVA was observed in this cohort of patients (Table 1).

### 2.3. Stargardt Disease

Schwartz et al. described the outcomes of 9 Stargardt’s disease patients treated in phase I/II study (median age 50 years, range 20–71) [21,22,23]. The patients were treated with an hESC-derived RPE cellular suspension obtained from a hESC line (MA09) and delivered into the subretinal space at the transition zone between healthy and atrophic retina following a vitrectomy. Patients underwent systemic low dose immunosuppression 1 week before surgery and up to 12 weeks afterwards. The authors report no adverse event resulting from the cellular therapy. However, 3 eyes developed cataracts that required surgery and 1 eye developed severe vitreous inflammation consistent with acute postoperative endophthalmitis that resolved in 2 months with antibiotic injection. Among the 8 STGD patients who had BCVA assessed at 6 months, 3 eyes improved by at least 15 letters, 4 eyes remained stable, and 1 eye decreased by 11 letters. At 12 months follow-up, 3 STGD patients improved by at least 15 letters, 3 were stable, and 1 had a decrease of more than 10 letters.

Mehat et al. described the outcomes of 12 patients with advanced Stargardt’s disease treated in a phase I/II open-label dose-escalation trial [27]. For each participant, the poorer-seeing eye was selected for intervention and the better-seeing contralateral eye served as an untreated control. The selected eyes underwent a 3-port pars plana vitrectomy followed by the injection of 0.2 mL Hartman’s solution through a 41G cannula to establish a target tissue plane. Eventually, an hESC line MA09-derived RPE cellular suspension was injected through a 38G subretinal cannula into the blebs of Hartmann’s solution at the transition zone between healthy and atrophic retina. To decrease the risk of immune rejection, oral immunosuppressive therapy was started 1 week before and continued for 12 weeks after transplantation. The authors reported no adverse events resulting from the cellular therapy. Adverse events related to immunosuppression occurred in 5 participants. No evidence of acute immune rejection was identified. Only 1 participant developed a nonpigmented, noncontractile epiretinal membrane over the injection site at month 6. This caused no measurable effect on visual function. Furtheremore, 2 participants reported visual floaters due to focal pigmented deposits in the vitreous cavity.

Although focal areas of subretinal hyperpigmentation developed in all participants in a dose-dependent manner, borderline improvement in BCVA in 4 participants was either not sustained or matched by a similar improvement in the untreated contralateral eye. Microperimetry demonstrated no evidence of benefit at 12 months in all 12 participants. In one instance, at the highest dose, localized retinal thinning and reduced sensitivity in the area of hyperpigmentation revealed the potential for harm. Participant-reported quality of life using the 25-item National Eye Institute Visual Function Questionnaire indicated no significant change.

Li et al. described the outcomes of 7 patients with Stargardt’s disease, aged 19–27 years, who were treated in a phase 1 clinical trial [28]. For each participant, the poorer-seeing eye was selected for intervention; the better-seeing contralateral eye served as an untreated control. The selected eye underwent a three-port pars plana vitrectomy followed by injection of a small amount of saline solution through a 41G cannula to detach the temporal retina. Eventually, 10^5^ hESC-derived RPE cells suspended in 100 μL volume were injected into the macular sub-retinal space followed by injection of silicon oil as tamponade. To inhibit detrimental immune reactions immunosuppression was administered 1 week before and continued up to 12 weeks after surgery. The authors report that neither severe local nor systemic complications occurred. However, 1–2 months after the operation, 2 patients had a transiently high intraocular pressure ranging from 26 to 32 mmHg, which was relieved by eye drops and cured after silicone oil removal. All of the operated eyes had transiently increased or had stable visual function 1–4 months after transplantation. At the last follow-up visit, 2 of the 7 eyes showed visual function loss. However, 1 of them showed a stable visual acuity when compared to the fellow eye.

Sung et al. discussed the long-term safety and tolerability of subretinal transplantation of hESC-derived RPE in Asian Stargardt disease patients [29]. The group conducted a non-randomized clinical trial including three patients with Stargardt macular disease. The eye with the poorest vision was selected in each patient to undergo pars plana vitrectomy and subretinal injection of a suspension of hESC-derived RPE cells. To *prevent* immune rejection, immunosuppression was achieved with tacrolimus and mycophenolate mofetil. No serious adverse events occurred in the three years follow up. While 1 patient showed BCVA improvement, the others maintained a stable BCVA (Table 1).

## 3. Discussion

### 3.1. Cellular Source of RPE Cells

#### 3.1.1. Induced Pluripotent Stem Cells

In 2006, Yamanaka et al. described the possibility to reprogram differentiated murine embryonic fibroblasts into iPSCs in culture following treatment with a cocktail of four main transcriptor factors, namely Oct3/4, Sox2, c-Myc, and Klf4 [31]. The following year, the same teamgenerate iPSCs from human somatic cells were treated following the same technique [32].

In culture, iPSCs are able to self-renew and differentiate towards any cell type of the body, and importantly, since they can be generated from adult tissues each individual, in theory, could have their autologous iPSC line. Thanks to these features, iPSCs hold a huge potential and are a great promise for regenerative medicine. Moreover, the original technique for dedifferentiation of somatic cells into iPSCs has been improved. This allowed the achievement of a better safety profile by minimizing genomic instability and tumorigenic potential [12,33].

iPSCs are a potentially unlimited source of RPE cells.

To date, most differentiation protocols generate hiPSC-derived RPE cells by spontaneous or directed differentiation. Spontaneous differentiation is labor intensive and highly variable across different iPSC lines [34]. Directed differentiation protocols can significantly increase yields in hiPS-RPE cell production [35,36,37,38]. However, they often requires time-consuming preparation protocols and are incompatible with cell therapy due to the use of animal-derived undefined products [39,40]. Very recently, safe and efficient protocols for hiPS-derived-RPE clinical-grade cells were described [41,42]. The advantage of iPSCs is that they can be collected easily and produced at a relatively reasonable price. They can be autologous, thus preventing rejection issues, and less ethical problems arise from their collection when compared to ESCs. The disadvantages include the mutation load of iPS cells, which pass through several stresses and long culture periods, and potential safety issues related to this [38]. These cells do indeed show inconstant ability to differentiate into the specific cell type required. When cells are autologous, genome editing would be required in patients with genetic mutations. On the other hand, when cells are allogenic, we need to consider that rejection may spoil long term results. Therefore, HLA matching and possibly immunosuppression would be needed (Table 2).

#### 3.1.2. Embryonic Stem Cells

ESCs are obtained from the internal mass of the preimplantation blastocyst [43]. ESCs just like iPSCs, exhibit indefinite self-renewal and ability to differentiate into all the different somatic cell lineages [44]. Their use is prohibited in several countries and their gathering and usage raise many ethical concerns [45].

ESCs need to be guided into their differentiation in order to commit as retinal cell progenitors. The process takes grossly the same modalities seen for iPSCs and implies exposure to transcription factors in cell cultures [35].

These cells have a high proliferative capacity. This is an advantage because it ensures a sustained renewal of the RPE. However, it exposes patients to the chance of developing tumors, particularly teratomas. Despite these risks, preclinical and clinical studies have shown a satisfactory safety profile [46] (Table 2).

#### 3.1.3. Transplant Type

The success of the replacement procedure does not solely rely on the isolation of the best suited cell population in adequate amount.

A key point is the capability to properly deliver the cellular pool collected in order to maximize the chances of engraftment and regeneration of the original structural and functional architecture of the tissue considered.

RPE replacement has historically relied on two main approaches in this sense: Injection of cell suspensions in the subretinal space and RPE patch transplant.

### 3.2. Cellular Suspensions

Injecting a cell suspension into the subretinal space has been shown to be feasible and safe as a technique. However, the preliminary results of the studies where this technique was adopted showed that the efficiency of the regeneration process and the survival of RPE cell pool is limited.

There is an array of hypotheses that could explain this, however not one provides conclusive explanation. What we know is that physiologically RPE cells form in nature polarized and tightly sealed layers of cells in close relationship with the overlying layer of PRC and the underlying Bruch’s membrane.

Restoring this three-dimensional organization in a diseased eye is a tall order. RPE cell survival relies on the adherence and the bonds cells are able to establish with the underlying collagenous matrix, which is nevertheless altered in disease [47]. The same collagenous matrix guides the polarization process which may therefore be altered in this condition [47]. The result is limited RPE polarization, unsatisfactory survival, and hence poor tissue function restoration (Table 3).

### 3.3. Patch Transplantation

The alternative approach for delivering RPE cells is to transfer them directly as a monolayer of polarized cells on a matrix acting as a scaffold.

This improves polarity and survival by substituting itself to the dysfunctional Bruch’s membrane which may be found on the site of RPE atrophy [48].

Many different materials have been studied, and they are most likely a key component in the success of the transplant. The scaffolding membrane materials proposed range from natural to synthetic fiber, from biodegradable to non-biodegradable [49].

The materials tested in the different trials are mainly PET polyester membranes covered with vitreonectin, Parylene C, as well as electrospun nanofiber membranes such as silk, polycaprolactone, polyamide, polyethylene glycol, and polymethyl methacrylate-co-polyethylene glycol methacrylate.

Biodegradable ones include poly-L-lactic acid and poly-lactic-co-glycolic acid [50].

The role of the matrix chosen is fundamental for the final result because ideally, one should choose a slowly biodegradable matrix which gives enough time for engraftment to occur before being broken down. Another important consideration is the tolerability of the matrix since it should not induce foreign body reaction but ideally promote immune modulation and restoration of the immune-privileged environment. This is a pivotal concept particularly when dealing with allogeneic transplant of partially HLA matched or unmatched donor RPE.

The results of the studies published so far suggest the safety and tolerability of this approach, better functional results, and longer RPE survival.

The main disadvantage of this strategy is the need to perform a more invasive surgical delivery of the graft. This is particularly true when the area that needs replacing is large (Table 3).

### 3.4. Delivery Options

Cellular suspensions are commonly delivered with a subretinal injection performed with a small (i.e., 38G) cannula in the transition zone between healthy and atrophic retina [23]. This technique carries the advantage of being easy and safe avoiding the need for a wide posterior retinotomy. Recently, a different subretinal delivery was evaluated in a Phase I/IIa clinical study in patients with advanced dry AMD and geographic atrophy (GA) (NCT02286089). In the latter study, the authors investigated the possibility of delivering subretinal injections with a novel tool: the Orbit subretinal delivery system (SDS) [25]. The SDS was FDA approved in August 2020 for subretinal injections. It uses a flexible cannula designed to contour the globe and access the subretinal space, the main advantage being the possibility of reaching the posterior pole without the need for vitrectomy and retinotomy. The SDS uses a flexible cannula that conforms to the curvature of the eye and is used to cannulate the suprachoroidal space. Inside the cannula, there is an advanceable microneedle used to penetrate the choroid and provide access to the subretinal space [51].

**Figure 1:**
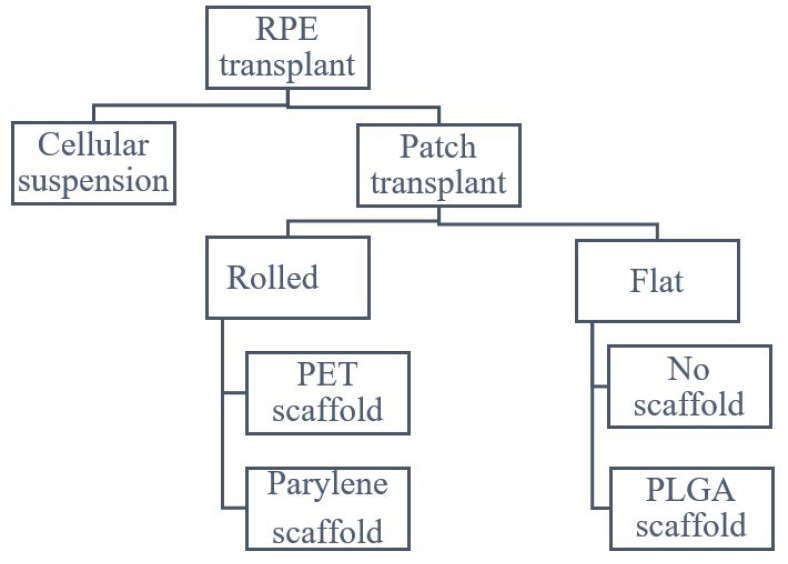
Scheme of possible RPE transplant delivery system.

On the other hand, patch transplant techniques need a wide posterior retinotomy to position the patch in place. Several devices have been proposed to accommodate the transplant in site. All patch implant surgeries share the same steps: a small gauge (38–41 G) cannula is used to elevate a bleb, followed by a subretinal hydrodissection with an infusion cannula and finally a retinotomy with vertical scissors is carried out. The main difference between the techniques is in the delivery tool: Kashani and Da Cruz used a delivery tool with a rolled RPE, while Mandai and Sharma used a tool to put in place an already flat RPE. Rolled RPE can be carried in a smaller device but is more difficult to locate in place due to the complexity of the unrolling maneuver (Figure 1).

The main complications of performing a posterior retinotomy are the development of epiretinal membrane (ERM) and proliferative vitreous retinopathy [52].

### 3.5. Current Drawbacks

Despite the previously discussed literature, many questions remain currently unaswered. Firstly, it is unknown if the disease pathogenesis may overcome the grafted RPE. In fact, the disease may proceed and affect the grafted RPE, downgrading this intervention as a temporary treatment and not a cure. In Stargardt’s disease, the “indirect secondary photoreceptor death” hypothesis is supported but not proved, and so if cell death was occurring prior to RPE involvement this would also undermine the value of RPE graft. Furthermore, if the indirect hypothesis is true, PRC would still carry the mutant ABCA4 transporter and the RPE graft would in turn become rapidly poisoned.

Secondly, functional reconnection of apical RPE on the transplanted patch and the photoreceptor outer segments has not been proved, while there is proof of anatomical proper positioning, safety, and tolerability. Indeed, there is a wide gap to reaching good efficacy of this technique. The functional integration of donor and recipient tissues is a crucial step.

## 4. Materials and Methods

The peer-reviewed literature was analyzed, and all relevant articles were selected. A literature search was conducted in April 2021 in Medline, the Cochrane Library, and the databases of clinical trials and was limited to studies published in English. The search strategy used the following MeSH terms and text words: retinal pigmented epithelium replacement, RPE transplant, iPS retinal transplant, ESC retinal transplant.

The initial search yielded 352 citations. Abstracts of meeting presentations were not included in the analysis because of their limited data.

The authors reviewed 247 abstracts and selected 114 articles of possible clinical relevance to review in full text. Of these, 11 were sufficiently clinically relevant.

## 5. Conclusions

Different approaches have been tested when researching the perfect balance between feasibility and clinical outcomes. So far, patch implants have reported good outcomes with a reasonable incidence of AE that will likely be reduced as the techniques become more refined. Cellular suspension approaches are interesting for the ease of the technical maneuvers they involve but seem less promising when the functional outcomes are concerned.

According to the result of this review, iPSCs vehiculated by patch transplant are the most promising option for RPE replacement therapy, as this technique allows to easily produce a considerable number of RPE-deriveded iPSCs and to preserve the physiological RPE polarization.

We highlight a few drawbacks. Firstly, RPE transplanted cell survival remains uncertain, as even when anatomical success is achieved, in vivo functionality is unknown. Secondly, there is lack of long-term follow-ups, which would be necessary to ensure the safety of these procedures.

RPE loss of function precedes photoreceptor death and represents a key target in the search for a therapeutic solution of AMD and Stargardt disease. Upcoming phase II and III trials will unveil the true potential of RPE transplant.

## Figures and Tables

**Table 1:** Resume of published clinical trials.

	Authors	N=	Transplant Type	Scaffold	Delivery Technique	Adverse Events	BCVA Improvement (ETDRS)	
**DRY AMD**	Schwartz et al.	9	hESC-derived suspension	n/a	Subretinal injection (38G) after vitrectomy	1 vitreous inflammation 1 mild ERM 1 cataract	15 letters in 4 eyes 11–14 letters in 3 eyes <10 letters in 3 eyes	
	Riemann et al. (poster)	16 (4 different cohorts)	hESC-derived RPE cells suspension	n/a	-PPV retinotomy -Orbit subretinal delivery system (SDS)	PPV group 2 severe ERM SDS group eyelid edema and subconjunctival hemorrhage	10–22 letters (cohort 4 n = 4)	
	Kashani et al.	5	hESC-derived RPE monolayers 3.5 mm × 6.25 mm	Parylene C	PPV and retinotomy		17 letters in 1 patient, none in the other 3	
**WET AMD**	Tezel et al.	12	adult human allogeneic RPE cells	n/a	PPV and subfoveal membranectomy with transplantation	6 graft fibrosis, 3 retinal detachments with PVR, 2 ERM, 1 graft migration	No change	
	Binder et al.	53 (39 transplanted, 14 controls)	Autologous RPE cellular suspension	n/a	PPV and subretinal injection	None	Better reading acuity and higher mfERG-response density than control subjects	
	Mandai et al.	1	autologous (iPSC) from skin fibroblasts 1.3 mm × 3 mm	no	PPV, membranectomy and patch implant		No change	
	Da Cruz et al.	2 with subretinal hemorrhage	hESC 6 mm × 3 mm	PET		1 retinal detachment	29 and 21 letters	
	Sugita et al.	5	HLA homozygote iPS cellular suspension		Subretinal injection (38G) after vitrectomy	2 mild rejections 1 ERM	No change	
**STGD1**	Schwartz et al.	9	hESC-derived RPE cells suspension		Subretinal injection after vitrectomy	3 cataracts 1 vitreous inflammation	At 6 months: At least 15 letters in 3 eyes, stable in 4 eyes and 1 eye lost 11 letters. At 12 months: At least 15 letters in 3 patients, stable in 3 patients, and 1 had a decrease of more than 10 letters.	
	Mehat et al.	12	hESC line MA09-derived RPE cellular suspension		Subretinal injection (38G) after vitrectomy	5 immunosuppression related events. 1nonpigmented and noncontractile epiretinal membrane. 2 visual floaters.	No change	
	Li et al.	7	hESC-derived RPE cells suspension		-PPV -injection of saline solution (41G) to detach the temporal retina. -cellular into the macular sub-retinal space.	2 transient increases in intraocular pressure (26–32 mmHg).	At 1–4 months: 7 eyes had transiently increased or stable visual function At the last follow-up visit: visual function loss in 2 eyes (1 of them stable when compared to the fellow eye).	
	Sung et al.	3	hESC-derived RPE cells suspension		Subretinal injection after vitrectomy		1 eye showed improvement, 1 eye showed stability	

PPV: pars plana vitrectomy, ERM: epiretinal membrane.

**Table 2:** Advantages and disadvantages of IPSCs and ESCs.

Induced Pluripotent Stem Cells		Embryonic Stem Cells	
Advantages	Disadvantages	Advantages	Disadvantages
Possibility to obtain multiple retinal cell populations	Genomic instability	Genomic stability	Forbidden in several countries
Easy and affordable collection and scale-up of production	Inconstant ability to differentiate into the desired cell linage	High self-renewal potential	High costs and lower availability
Possibility to perform both autologous and allogenic transplants	Rejection in allogenic transplants	Reduced rejection in allogenic transplants	Ethical issues related to their collection and use
No ethical issues			

**Table 3:** Advantages and disadvantages of cellular suspensions and patch transplantation.

Cellular Suspensions		Patch Transplantation	
Advantages	Disadvantages	Advantages	Disadvantages
Easy and safe delivery	Limited efficiency and regenerative potential	Satisfactory efficiency and regenerative potential	More complex and invasive delivery
	Self-exhaustion of the cell population	Prolonged survival of the graft	Possible foreign body reaction to matrices
	Lack of polarization of the derived RPE	Polarization of RPE cells	

## References

[1] Nguyen-Legros J., Hicks D. (2000). Renewal of photoreceptor outer segments and their phagocytosis by the retinal pigment epithelium. Int. Rev. Cytol..

[2] Strauß O. (2016). Pharmacology of the retinal pigment epithelium, the interface between retina and body system. Eur. J. Pharmacol..

[3] Sparrow J.R., Hicks D., Hamel C.P. (2010). The retinal pigment epithelium in health and disease. Curr. Mol. Med..

[4] Anderson D.H., Radeke M.J., Gallo N.B., Chapin E.A., Johnson P.T., Curletti C.R., Hancox L.S., Hu J., Ebright J.N., Malek G. (2010). The pivotal role of the complement system in aging and age-related macular degeneration: Hypothesis re-visited. Prog. Retin. Eye Res..

[5] Anderson D.H., Mullins R.F., Hageman G.S., Johnson L.V. (2002). A role for local inflammation in the formation of drusen in the aging eye. Am. J. Ophthalmol..

[6] Ida H., Tobe T., Nambu H., Matsumura M., Uyama M., Campochiaro P.A. (2003). RPE cells modulate subretinal neovascularization, but do not cause regression in mice with sustained expression of VEGF. Investig. Ophthalmol. Vis. Sci..

[7] Ao J., Wood J.P., Chidlow G., Gillies M.C., Casson R.J. (2018). Retinal pigment epithelium in the pathogenesis of age-related macular degeneration and photobiomodulation as a potential therapy?. Clin. Exp. Ophthalmol..

[8] Hanus J., Anderson C., Wang S. (2015). RPE necroptosis in response to oxidative stress and in AMD. Ageing Res. Rev..

[9] Datta S., Cano M., Ebrahimi K., Wang L., Handa J.T. (2017). The impact of oxidative stress and inflammation on RPE degeneration in non-neovascular AMD. Prog. Retin. Eye Res..

[10] Van Lookeren Campagne M., LeCouter J., Yaspan B.L., Ye W. (2014). Mechanisms of age-related macular degeneration and therapeutic opportunities. J. Pathol..

[11] Bhutto I., Lutty G. (2012). Understanding age-related macular degeneration (AMD): Relationships between the photoreceptor/retinal pigment epithelium/Bruch’s membrane/choriocapillaris complex. Mol. Asp. Med..

[12] Bracha P., Moore N.A., Ciulla T.A. (2017). Induced pluripotent stem cell-based therapy for age-related macular degeneration. Expert Opin. Biol. Ther..

[13] Mitter S.K., Song C., Qi X., Mao H., Rao H., Akin D., Lewin A., Grant M., Dunn W., Ding J. (2014). Dysregulated autophagy in the RPE is associated with increased susceptibility to oxidative stress and AMD. Autophagy.

[14] Ciulla T.A., Harris A., Kagemann L., Danis R.P., Pratt L.M., Chung H.S., Weinberger D., Garzozi H.J. (2002). Choroidal perfusion perturbations in non-neovascular age related macular degeneration. Br. J. Ophthalmol..

[15] Tsang S.H., Sharma T. (2018). Stargardt Disease. Adv. Exp. Med. Biol..

[16] Tanna P., Strauss R.W., Fujinami K., Michaelides M. (2017). Stargardt disease: Clinical features, molecular genetics, animal models and therapeutic options. Br. J. Ophthalmol..

[17] Idelson M., Alper R., Obolensky A., Yachimovich-Cohen N., Rachmilewitz J., Ejzenberg A., Beider E., Banin E., Reubinoff B. (2018). Immunological Properties of Human Embryonic Stem Cell-Derived Retinal Pigment Epithelial Cells. Stem Cell Rep..

[18] Kramer J., Chirco K.R., Lamba D.A. (2019). Immunological Considerations for Retinal Stem Cell Therapy. Adv. Exp. Med. Biol..

[19] Safety and Efficacy of Intravitreal Injection of Human Retinal Progenitor Cells in Adults with Retinitis Pigmentosa [Internet]. ClinicalTrials.gov Identifier: NCT03073733. NCT03073733.

[20] Singh R.K., Occelli L.M., Binette F., Petersen-Jones S.M., Nasonkin I.O. (2019). Transplantation of Human Embryonic Stem Cell-Derived Retinal Tissue in the Subretinal Space of the Cat Eye. Stem Cells Dev..

[21] Schwartz S.D., Hubschman J.-P., Heilwell G., Franco-Cardenas V., Pan C.K., Ostrick R.M., Mickunas E., Gay R., Klimanskaya I., Lanza R. (2012). Embryonic stem cell trials for macular degeneration: A preliminary report. Lancet.

[22] Schwartz S.D., Tan G., Hosseini H., Nagiel A. (2016). Subretinal Transplantation of Embryonic Stem Cell-Derived Retinal Pigment Epithelium for the Treatment of Macular Degeneration: An Assessment at 4 Years. Invest. Ophthalmol. Vis. Sci..

[23] Schwartz S.D., Regillo C.D., Lam B.L., Eliott D., Rosenfeld P.J., Gregori N.Z., Hubschman J.P., Davis J.L., Heilwell G., Spirn M. (2015). Human embryonic stem cell-derived retinal pigment epithelium in patients with age-related macular degeneration and Stargardt’s macular dystrophy: Follow-up of two open-label phase 1/2 studies. Lancet.

[24] Tezel T.H., Del Priore L.V., Berger A.S., Kaplan H.J. (2007). Adult retinal pigment epithelial transplantation in exudative age-related macular degeneration. Am. J. Ophthalmol..

[25] Sharma A., Jaganathan B.G. (2021). Stem Cell Therapy for Retinal Degeneration: The Evidence to Date. Biol. Targets Ther..

[26] Sugita S., Mandai M., Hirami Y., Takagi S., Maeda T., Fujihara M., Matsuzaki M., Yamamoto M., Iseki K., Hayashi N. (2020). HLA-Matched Allogeneic iPS Cells-Derived RPE Transplantation for Macular Degeneration. J. Clin. Med..

[27] Mehat M.S., Sundaram V., Ripamonti C., Robson A.G., Smith A.J., Borooah S., Robinson M., Rosenthal A.N., Innes W., Weleber R.G. (2018). Transplantation of Human Embryonic Stem Cell-Derived Retinal Pigment Epithelial Cells in Macular Degeneration. Ophthalmology.

[28] Li S.-Y., Liu Y., Wang L., Wang F., Zhao T.-T., Li Q.-Y., Xu H.W., Meng X.H., Hao J., Zhou Q. (2021). A phase I clinical trial of human embryonic stem cell-derived retinal pigment epithelial cells for early-stage Stargardt macular degeneration: 5-years’ follow-up. Cell Prolif..

[29] Sung Y., Lee M.J., Choi J., Jung S.Y., Chong S.Y., Sung J.H., Shim S.H., Song W.K. (2021). Long-term safety and tolerability of subretinal transplantation of embryonic stem cell-derived retinal pigment epithelium in Asian Stargardt disease patients. Br. J. Ophthalmol..

[30] Da Cruz L., Fynes K., Georgiadis O., Kerby J., Luo Y.H., Ahmado A., Vernon A., Daniels J.T., Nommiste B., Hasan S.M. (2018). Phase 1 clinical study of an embryonic stem cell-derived retinal pigment epithelium patch in age-related macular degeneration. Nat. Biotechnol..

[31] Takahashi K., Yamanaka S. (2006). Induction of pluripotent stem cells from mouse embryonic and adult fibroblast cultures by defined factors. Cell.

[32] Takahashi K., Tanabe K., Ohnuki M., Narita M., Ichisaka T., Tomoda K., Nakamura M., Sutou K., Osafune K., Yamanaka S. (2007). Induction of pluripotent stem cells from adult human fibroblasts by defined factors. Cell.

[33] Nakagawa M., Koyanagi M., Tanabe K., Takahashi K., Ichisaka T., Aoi T., Okita K., Mochiduki Y., Takizawa N., Yamanaka S. (2008). Generation of induced pluripotent stem cells without Myc from mouse and human fibroblasts. Nat. Biotechnol..

[34] Leach L.L., Croze R.H., Hu Q., Nadar V.P., Clevenger T.N., Pennington B.O., Gamm D.M., Clegg D.O. (2016). Induced Pluripotent Stem Cell-Derived Retinal Pigmented Epithelium: A Comparative Study Between Cell Lines and Differentiation Methods. J. Ocul. Pharmacol. Ther..

[35] Idelson M., Alper R., Obolensky A., Ben-Shushan E., Hemo I., Yachimovich-Cohen N., Khaner H., Smith Y., Wiser O., Gropp M. (2009). Directed differentiation of human embryonic stem cells into functional retinal pigment epithelium cells. Cell Stem Cell..

[36] Osakada F., Ikeda H., Sasai Y., Takahashi M. (2009). Stepwise differentiation of pluripotent stem cells into retinal cells. Nat. Protoc..

[37] Plaza Reyes A., Petrus-Reurer S., Padrell Sánchez S., Kumar P., Douagi I., Bartuma H., Aronsson M., Westman S., Lardner E., André H. (2020). Identification of cell surface markers and establishment of monolayer differentiation to retinal pigment epithelial cells. Nat. Commun..

[38] Michelet F., Balasankar A., Teo N., Stanton L.W., Singhal S. (2020). Rapid generation of purified human RPE from pluripotent stem cells using 2D cultures and lipoprotein uptake-based sorting. Stem Cell Res. Ther..

[39] Leach L.L., Buchholz D.E., Nadar V.P., Lowenstein S.E., Clegg D.O. (2015). Canonical/-Catenin Wnt Pathway Activation Improves Retinal Pigmented Epithelium Derivation From Human Embryonic Stem Cells. Invest. Ophthalmol. Vis Sci..

[40] Buchholz D.E., Pennington B.O., Croze R.H., Hinman C.R., Coffey P.J., Clegg D.O. (2013). Rapid and Efficient Directed Differentiation of Human Pluripotent Stem Cells into Retinal Pigmented Epithelium. Stem Cells Transl. Med..

[41] Sharma R., Khristov V., Rising A., Jha B.S., Dejene R., Hotaling N., Li Y., Stoddard J., Stankewicz C., Wan Q. (2019). Clinical-grade stem cell-derived retinal pigment epithelium patch rescues retinal degeneration in rodents and pigs. Sci. Transl. Med..

[42] Limnios I.J., Chau Y.-Q., Skabo S.J., Surrao D.C., O’Neill H.C. (2021). Efficient differentiation of human embryonic stem cells to retinal pigment epithelium under defined conditions. Stem Cell Res. Ther..

[43] Thomson J.A., Itskovitz-Eldor J., Shapiro S.S., Waknitz M.A., Swiergiel J.J., Marshall V.S., Jones J.M. (1998). Embryonic stem cell lines derived from human blastocysts. Science.

[44] Jia J., Zheng X., Hu G., Cui K., Zhang J., Zhang A., Jiang H., Lu B., Yates J., Liu C. (2012). Regulation of pluripotency and self- renewal of ESCs through epigenetic-threshold modulation and mRNA pruning. Cell.

[45] Shenfield F. (2005). Semantics and ethics of human embryonic stem-cell research. Lancet.

[46] Lu B., Malcuit C., Wang S., Girman S., Francis P., Lemieux L., Lanza R., Lund R. (2009). Long-term safety and function of RPE from human embryonic stem cells in preclinical models of macular degeneration. Stem Cells.

[47] Sugino I.K., Sun Q., Cheewatrakoolpong N., Malcuit C., Zarbin M.A. (2014). Biochemical restoration of aged human Bruch’s membrane: Experimental studies to improve retinal pigment epithelium transplant survival and differentiation. Dev. Ophthalmol..

[48] Delplace V., Payne S., Shoichet M. (2015). Delivery strategies for treatment of age-related ocular diseases: From a biological understanding to biomaterial solutions. J. Control. Release Off. J. Control Release Soc..

[49] Xiang P., Wu K.-C., Zhu Y., Xiang L., Li C., Chen D.-L., Chen F., Xu G., Wang A., Li M. (2014). A novel Bruch’s membrane-mimetic electrospun substrate scaffold for human retinal pigment epithelium cells. Biomaterials.

[50] O’Neill H.C., Limnios I.J., Barnett N.L. (2020). Advancing a Stem Cell Therapy for Age-Related Macular Degeneration. Curr. Stem Cell Res. Ther..

[51] De Smet M.D., Lynch J.L., Dejneka N.S., Keane M., Khan I.J. (2018). A Subretinal Cell Delivery Method via Suprachoroidal Access in Minipigs: Safety and Surgical Outcomes. Invest. Ophthalmol. Vis. Sci..

[52] Sharma R., Bose D., Maminishkis A., Bharti K. (2020). Retinal Pigment Epithelium Replacement Therapy for Age-Related Macular Degeneration: Are We There Yet?. Annu. Rev. Pharmacol. Toxicol..

